# Surgery for craniopharyngioma

**DOI:** 10.3171/2026.8.FOCVID2565

**Published:** 2026-10-01

**Authors:** Amir R. Dehdashti, Luigi M. Cavallo, Paul A. Gardner, Viviane Tabar, Mustafa K. Baskaya, James J. Evans, Henry Dufour

**Affiliations:** 1 Department of Neurosurgery, North Shore University Hospital, Northwell Health, Donald and Barbara Zucker School of Medicine at Hofstra/Northwell, Manhasset, New York;; 2 Division of Neurosurgery, Department of Neurosciences, Reproductive and Odontostomatological Sciences, Università degli Studi di Napoli Federico II, Naples, Italy;; 3 Department of Neurosurgery, NYU Langone, New York;; 4 Department of Neurosurgery, Memorial Sloan-Kettering Cancer Center, New York, New York;; 5 Department of Neurological Surgery, School of Medicine and Public Health, University of Wisconsin, Madison, Wisconsin;; 6 Department of Neurological Surgery, Thomas Jefferson University, Philadelphia, Pennsylvania; and; 7 Department of Neurosurgery, Hôpital de la Timone, AP-HM, Aix-Marseille University, Marseille, France

## INTRODUCTION

Craniopharyngiomas, benign epithelial tumors that arise in the sellar and suprasellar regions of the brain, account for approximately 2%–5% of all intracranial tumors. Despite their benign histology, these tumors are clinically challenging because of their proximity to critical neurovascular structures. Two major pathological subtypes are recognized: adamantinomatous craniopharyngioma, more common in children, and papillary craniopharyngioma, which predominantly affects adults. During the past 2 decades, major advances in molecular biology, imaging, neurosurgical techniques, radiotherapy, and targeted therapies have significantly transformed the management of these complex tumors.^1^

Historically, radical resection was considered the standard treatment for craniopharyngiomas. However, aggressive surgery was frequently associated with substantial morbidity, including visual impairment, endocrine dysfunction, hypothalamic injury, obesity, cognitive decline, and reduced quality of life. Increasing recognition of these complications has shifted modern management toward a more balanced and individualized treatment strategy that prioritizes long-term functional outcomes in addition to tumor control.

While gross-total resection should remain as the treatment of choice when safely feasible, there are alternative strategies that can improve surgical outcomes.

One of the most important advances in the surgical management of craniopharyngiomas has been the development of minimally invasive endoscopic endonasal approaches that provide improved visualization of the suprasellar region while minimizing brain retraction and surgical trauma.^2,3^ Recent studies have demonstrated favorable outcomes with endoscopic surgery, including improved vision recovery, lower postoperative morbidity, and shorter hospital stays. Nevertheless, surgical planning remains highly individualized, as tumor extension, hypothalamic involvement, and patient age continue to influence the choice of operative approach.

Very large tumors can benefit from combined endonasal and transcranial approaches to address significant lateral and superior extension of the tumor.

Another major shift has been the increasing use of subtotal/near-total resection followed by adjuvant radiotherapy. This strategy aims to reduce the risks associated with aggressive surgery while maintaining effective tumor control. Modern radiation techniques such as proton beam therapy, intensity-modulated radiotherapy, and stereotactic radiosurgery have improved the precision of treatment delivery, thereby reducing radiation toxicity. The use of proton therapy, in particular, has gained attention in pediatric patients because of its potential to minimize long-term neurocognitive and endocrine complications.

In recent years, molecular and genetic discoveries have provided new insights into the pathogenesis of craniopharyngiomas and opened the door to targeted therapies. Adamantinomatous craniopharyngiomas are commonly associated with mutations in the *CTNNB1* gene, leading to activation of the Wnt/β-catenin signaling pathway. In contrast, papillary craniopharyngiomas frequently harbor the BRAF V600E mutation. This discovery is particularly significant because it has enabled the use of targeted BRAF and MEK inhibitors in selected patients. Clinical reports have shown dramatic tumor shrinkage and symptom improvement following treatment with these agents, especially in recurrent or unresectable papillary craniopharyngiomas. These findings represent a major paradigm shift, as medical therapy may now reduce the need for extensive surgery or radiation therapy in selected cases.

Current research increasingly emphasizes quality of life and functional preservation as key treatment outcomes. Long-term survivors of craniopharyngioma often experience endocrine deficiencies, hypothalamic obesity, psychosocial challenges, and cognitive impairments that require lifelong multidisciplinary care. The patient-centered approach reflects the growing understanding that successful treatment involves not only tumor control but also optimization of neurological, endocrine, and psychosocial health.

In conclusion, the management of craniopharyngiomas has evolved significantly with advances in minimally invasive surgery, precision radiotherapy, molecular diagnostics, and targeted therapies. The modern paradigm is well demonstrated in this issue of *Neurosurgical Focus: Video* with robust and highly selected peer-reviewed video illustrations of several categories from experienced skull base surgery centers around the globe. It offers a modern, comprehensive, and diverse surgical strategy for different types of craniopharyngiomas in pediatric and adult patients. The videos in this issue address pediatric craniopharyngiomas (endonasal, combined, and transcranial approaches), adult craniopharyngiomas (endonasal endoscopic, combined endoscopic and transcranial, and purely transcranial approaches), and topics related to technical and management considerations, including intraoperative management.

## Disclosures

P. Gardner reported personal fees from Mizuho, Sutter, Medtronic, and Peter Lazic outside the submitted work. J. Evans reported royalties from Mizuho outside the submitted work.
